# Bioengineered kidney tubules efficiently excrete uremic toxins

**DOI:** 10.1038/srep26715

**Published:** 2016-05-31

**Authors:** J. Jansen, M. Fedecostante, M. J. Wilmer, J. G. Peters, U. M. Kreuser, P. H. van den Broek, R. A. Mensink, T. J. Boltje, D. Stamatialis, J. F. Wetzels, L. P. van den Heuvel, J. G. Hoenderop, R. Masereeuw

**Affiliations:** 1 Department of Pharmacology and Toxicology, Radboud university medical center, Radboud Institute for Molecular Life Sciences, Nijmegen, The Netherlands; 2 Physiology, Radboud university medical center, Radboud Institute for Molecular Life Sciences, Nijmegen, The Netherlands; 3 Department of Pediatrics, Radboud university medical center, Nijmegen, The Netherlands; 4 Division of Pharmacology, Utrecht Institute for Pharmaceutical Sciences, Utrecht, The Netherlands; 5 Cluster for Molecular Chemistry, Institute for Molecules and Materials, Radboud University, Nijmegen, The Netherlands; 6 Department of Biomaterials Science and Technology, MIRA Institute for Biomedical Technology and Technical Medicine, University of Twente, The Netherlands; 7 Nephrology, Radboud university medical center, Nijmegen, The Netherlands; 8 Department of Pediatric Nephrology & Growth and Regeneration, Catholic University Leuven, Leuven, Belgium

## Abstract

The development of a biotechnological platform for the removal of waste products (e.g. uremic toxins), often bound to proteins in plasma, is a prerequisite to improve current treatment modalities for patients suffering from end stage renal disease (ESRD). Here, we present a newly designed bioengineered renal tubule capable of active uremic toxin secretion through the concerted action of essential renal transporters, *viz.* organic anion transporter-1 (OAT1), breast cancer resistance protein (BCRP) and multidrug resistance protein-4 (MRP4). Three-dimensional cell monolayer formation of human conditionally immortalized proximal tubule epithelial cells (ciPTEC) on biofunctionalized hollow fibers with maintained barrier function was demonstrated. Using a tailor made flow system, the secretory clearance of human serum albumin-bound uremic toxins, indoxyl sulfate and kynurenic acid, as well as albumin reabsorption across the renal tubule was confirmed. These functional bioengineered renal tubules are promising entities in renal replacement therapies and regenerative medicine, as well as in drug development programs.

Chronic renal failure (CRF) is a severe health problem with a high morbidity and mortality rate as adequate therapy is currently not available. Impaired renal function results in the accumulation of various endogenous uremic metabolites (i.e. uremic toxins), which are associated with a broad range of pathologies that constitute the uremic syndrome^1^,^2^,^3^. The preferred treatment option for end-stage renal disease (ESRD) is organ transplantation, however, the worldwide organ shortage is profound and many patients experience graft failure^4^,^5^. Chronic dialysis (hemodialysis or peritioneal dialysis) is currently the best alternative treatment option, which is widely applied and efficient in removal of small water-soluble and middle molecular weight molecules, but it insufficiently removes larger and protein-bound uremic toxins^6^,^7^. The latter class comprises end-metabolites that originate from dietary breakdown amino acids, such as tyrosine, phenylalanine and l-tryptophan, for which their pathological role in the progression of the uremic syndrome has gained substantial interest in the last decade^6^.

The majority of dietary protein derived l-tryptophan is metabolized to l-kynurenine, which in turn can be converted into kynurenic acid. Accumulation of kynurenic acid was found to correlate with several symptoms of uremia, including neurological disturbances, lipid metabolism disorder and anemia^8^. Tryptophan can also be metabolized by intestinal bacteria into indoles, which are processed further in the liver into indoxyl sulfate, indole-3 acetic acid and indoxyl-β-D-glucuronide^9^. Indoxyl sulfate as well as the tyrosine end-metabolites *p*-cresyl sulfate and *p*-cresyl glucuronide are associated with cardiovascular disease development via the activation of leukocytes, reactive oxygen species (ROS) production and their interaction with the vascular endothelium^10^,^11^,^12^. Furthermore, the *p*-cresol derivates demonstrated cytotoxicity and a pro-inflammatory response in renal epithelial cells^13^, possibly contributing to CRF progression. Also, drug disposition is altered in patients with CRF, not only because of diminished kidney function but also due to a direct inhibition of drug transporters and drug-metabolism enzymes by uremic toxins^14^,^15^.

The residual renal function in ESRD patients is not only associated with the remaining glomerular filtration capacity, but also the ability of the proximal nephron segment to actively secrete protein-bound uremic toxins into the pro-urine^16^,^17^. To this end, proximal tubule epithelial cells (PTEC) are equipped with a broad range of transport proteins that accept a wide range of xenobiotics, including exogenous compounds such as drugs, and endogenous (waste) metabolites. At the basolateral membrane the organic anion transporters 1 (OAT1; *SLC22A6*) and −3 (OAT3; *SLC22A8*) of the solute carrier family (SLC), are highly efficient in the uptake of the anionic uremic toxins such as the l-tryptophan, tyrosine and phenylalanine end-metabolites, shifting their protein binding to the free fraction^8^,^18^,^19^. At the luminal side, the ATP binding cassette (ABC) transporters breast cancer resistance protein (BCRP; *ABCG2*) and the multidrug resistance-associated proteins 2 and −4 (MRP2/4; *ABCC2/4*) are involved in their urinary secretion^20^.

As the removal of protein-bound uremic toxins via PTEC is associated with better patients survival, the engineering of a bioartificial kidney (BAK) containing PTEC cultured on hollow fiber membranes (HFM) could be a promising platform to advance uremic toxin clearance. This was the focus of the current study using human conditionally immortalized PTEC (ciPTEC), expressing endogenously a broad range of functional transporters associated with uremic toxin handling^21^,^22^. Recently, complemented with OAT1 and −3 that are generally lost in PTEC upon culturing^23^. The cells carry the temperature-sensitive mutant U19tsA58 of SV40 large T antigen (SV40T) and the essential catalytic subunit of human telomerase (hTERT), allowing the cells to proliferate at the permissive low temperature of 33 °C and differentiate to mature PTEC at 37 °C, and maintenance of telomere length preventing replicative senescence, respectively. This resulted in stable cell lines that could be maintained over a long period of time, and a valuable tool for studying renal clearance processes as required for BAK engineering.

In the present study, we demonstrate the development of functional bioengineered renal tubules that efficiently clear protein-bound anionic uremic toxins. First, the essential role of in- and efflux transporters in the removal of uremic toxins was studied in flat monolayers. Subsequently, three dimensional, polarized, ciPTEC monolayers on biofunctionalized polyethersulfone hollow fiber membranes were developed. Finally, as a crucial next step in BAK engineering, the secretory clearance of human serum albumin-bound indoxyl sulfate and kynurenic acid was confirmed, as well as albumin reabsorption.

## Results

### Uremic toxins inhibit OAT1 and OAT3 activity at clinically relevant concentrations

**Figure 1:**
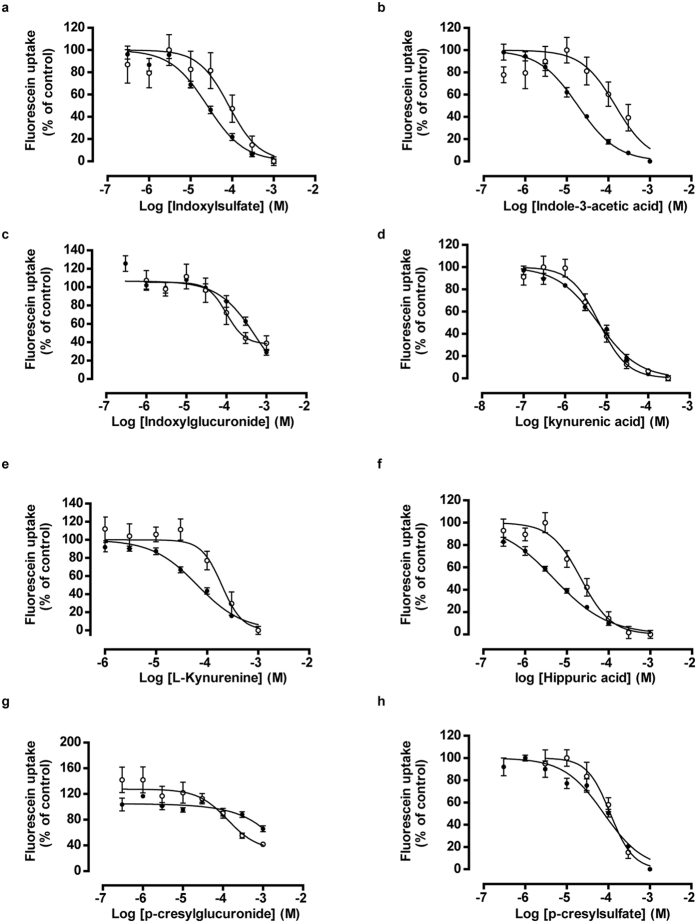
Concentration-dependent inhibition of OAT1- and OAT3-mediated fluorescein uptake by anionic uremic toxins. (**a–h**) A concentration range of eight uremic toxins were exposed to matured ciPTEC-OAT1 (•) and -OAT3 (o) in the presence of 1 μM fluorescein, a known OAT model substrate. The intracellular fluorescent signal was detected by measuring samples at excitation wavelength 485 nm and emission wavelength 535 nm. Blank data were subtracted and data were normalized to control (absence of uremic toxin). Nonlinear regression analysis was performed using Graphpad Prism 5.02. Data are shown as mean ± S.E.M. of three independent experiments performed, at least, in duplicate.

To evaluate the role of uptake transporters in uremic toxin handling, a panel of eight anionic uremic toxins was selected to study their affinity to inhibit OAT1- and OAT3-mediated uptake in ciPTEC. These toxins were selected based on their structure and potential PTEC-mediated urinary secretion, and their previously suggested association with complications of CRF^13^,^14^,^15^,^19^. The OAT1 and −3 model substrate fluorescein was used to evaluate transporter function^23^. A concentration-dependent inhibition of fluorescein uptake was shown for all anionic uremic toxins tested (Fig. 1), with most potent interactions found for kynurenic acid and hippuric acid (Fig. 1d,f, resp. and Table 1). The inhibitory potencies of the toxins, as reflected by their IC_50_ values (Table 1), were higher for OAT1 compared to OAT3, except for *p*-cresylglucuronide.

### The renal excretion of indoxyl sulfate and kynurenic acid is OAT1-, BCRP- and MRP4-mediated

**Figure 2:**
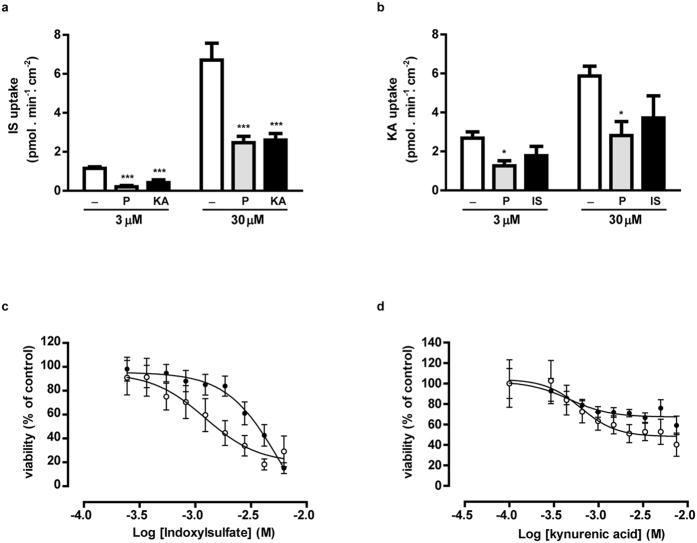
Handling of indoxyl sulfate and kynurenic acid by OAT1, BCRP and MRP4 in flat monolayers. In matured ciPTEC-OAT1, a concentration-dependent uptake of (**a**) IS and (**b**) KA (white) was shown using LC-MS/MS analysis of the uremic toxins. In the presence of probenecid (P, grey), the uptake of both toxins was attenuated. Moreover, the IS uptake was inhibited in the presence of KA (100 μM; black), and vice versa. The role of BCRP and MRP4 in the efflux of IS and KA was shown using an MTT assay. The experiment was performed in the absence (•) or presence (◦) of efflux pump inhibitors (KO143 (10 μM) and MK571 (5 μM)). Nonlinear regression analysis was performed using Graphpad Prism 5.02. Data are shown as mean ± S.E.M. of three independent experiments performed in triplicate. *p < 0.05, ***p < 0.001 using one-way ANOVA analysis followed by Dunnett’s multiple comparison test.

Indoxyl sulfate and kynurenic acid were selected for further studies using the ciPTEC-OAT1 model, as both showed a strong OAT1-mediated inhibition and have been associated severely with ESRD progression and its related complications^8^,^15^,^24^,^25^. Next to their inhibitory potency, we studied active PTEC transport processes. A dose-dependent uptake of indoxyl sulfate by ciPTEC-OAT1 monolayers was observed (Fig. 2a, 1.2 ± 0.1 and 6.7 ± 0.9 pmol.min^−1^.cm^−2^, at 3 and 30 μM respectively), which was sensitive to probenecid inhibition (by 83 ± 6 and 63 ± 5% for 3 and 30 μM, respectively; p < 0.001), a classical OAT inhibitor^26^. Indoxyl sulfate uptake was also inhibited by kynurenic acid (100 μM; by 64 ± 13 and 61 ± 5% for 3 and 30 μM, respectively; p < 0.001), while kynurenic acid uptake by OAT1 was not significantly affected by indoxyl sulfate (100 μM), most likely due to a higher affinity of kynurenic acid for the transporter than indoxyl sulfate^8^. But dose-dependent kynurenic acid uptake (Fig. 2b, 2.7 ± 0.3 and 5.9 ± 0.5 pmol.min^−1^.cm^−2^ for 3 and 30 μM, respectively) in ciPTEC-OAT1 was also sensitive to probenecid (53 ± 10 and 52 ± 12% inhibition, respectively; p < 0.05).

Using a vesicular transport assay for evaluating the activities of BCRP and MRP4^14^, Mutsaers *et al.* showed previously that indoxyl sulfate and kynurenic acid are potent inhibitors of both efflux pumps. Moreover, the intrinsic PTEC toxicity of the uremic toxins was demonstrated by their ability to reduce renal metabolic capacity and to increase free radical production in proximal tubule epithelial cells^15^,^19^. Here, the role of BCRP and MRP4 in indoxyl sulfate and kynurenic acid detoxification was studied further using a cell viability assay (Fig. 2c,d). CiPTEC showed to be slightly more sensitive to both uremic toxins when BCRP and MRP4 were inhibited by KO143 and MK571 (resp.), as demonstrated by decreased *TC*_50_ values (indoxyl sulfate: 2.0 ± 0.7 mM; kynurenic acid: 9.0 ± 3.0) compared to the *TC*_50_ values in the absence of inhibitors, though not significant (indoxyl sulfate: 3.6 ± 0.6 mM; kynurenic acid: not applicable). To support the importance of the combined effort of in- and efflux transport pathways especially in indoxyl sulfate detoxification, parent ciPTEC models lacking OAT1 demonstrated enhanced resistance against indoxyl sulfate in the presence of efflux pump inhibitors (*TC*_50_ 3.0 ± 0.5 mM).

### The bioengineered renal tubule shows a three dimensional, tight and differentiated epithelial monolayer

**Figure 3:**
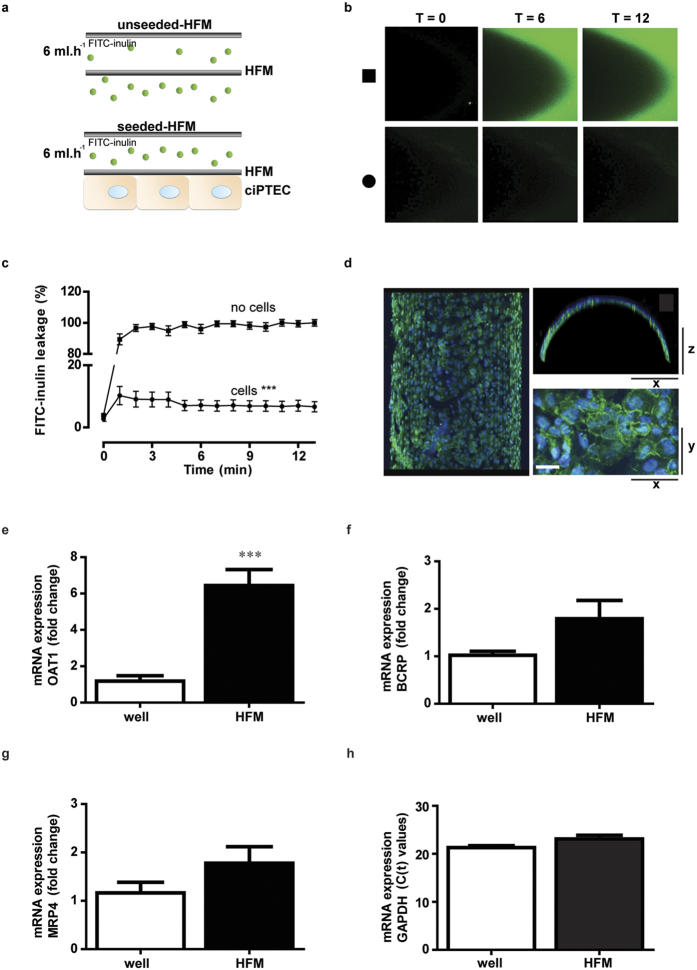
Bioengineered renal tubules show transepithelial barrier function and polarized characteristics. FITC-inulin leakage was measured in matured ciPTEC-OAT1 seeded on coated HFM. (**a**) Schematic presentation of the experimental set-up in the absence (upper panel) or presence (lower panel) of cells. (**b**) Representative eal-time images of the two different conditions are shown; unseeded (square) and seeded (circle) HFM. In the absence of cells, a bright green fluorescent signal was detected at the apical membrane, whereas in the presence of cells the signal was clearly attenuated. Representative real-time images of the two different conditions were shown. (**c**) Semi-quantification of FITC-inulin diffusion in the absence (square) and the presence of ciPTEC-OAT1 (circle) on HFM. Seeded HFM showed significantly less FITC-inulin leakage demonstrating monolayer tightness. From each single replicate 4 different regions in focus were analyzed. (**d**) The expression of ZO-1 (green) was demonstrated in a homogenous cell monolayer cultured on HFM. Scale bar: 10 μm. (**e–g**) The OAT1, BCRP and MRP4 mRNA expression levels were investigated in matured ciPTEC-OAT1 cultured in well plates and on HFM. Gene expression levels were normalized to expression levels of the reference gene GADPH (**h**) and were expressed as fold increase when cultured on HFM (black) compared to matured cells in well plates (white). Data are shown as mean ± S.E.M. of three independent experiments performed, at least, in duplicate. ***p < 0.001, using an unpaired *t* test.

Further development of a biotechnological platform for the removal of protein-bound waste products requires an optimal three-dimensional configuration, as a two-dimensional system poorly predicts renal xenobiotic handling. We recently successfully developed a three-dimensional bioengineered tubule system^27^, which we here optimized and determined monolayer integrity by analyzing paracellular diffusion prior to transport experiments. The transepithelial barrier function was measured using a custom-made flow system and inulin as a leakage marker labeled with fluorescein isothiocyanate (FITC), allowing live imaging of hollow fiber membranes (HFM). Differentiated ciPTEC-OAT1 on double-coated HFM were compared to unseeded coated HFM (Fig. 3a). Within 1 min of perfusion, unseeded-HFM showed a sustained leakage compared to the fully PTEC covered HFM (Fig. 3b,c, no cells: 89 ± 4% *vs.* cells: 10 ± 3%; p < 0.001). This effect remained stable until the end of the perfusion experiment, thereby confirming the formation of a three-dimensional, efficient and stable transepithelial barrier by ciPTEC-OAT1 on HFM. To further elucidate polarization characteristics of ciPTEC-OAT1 monolayers when cultured in a 3-dimensional (3D) HFM environment, the barrier function was also evaluated in 2D monolayers cultured on Transwell® filter inserts. When exposed to FITC-inulin, monolayers on inserts showed a limited barrier function of 20 ± 4% (p = 0.08) compared to unseeded filters (Fig. S1), confirming poor monolayer formation of ciPTEC-OAT1 using 2D systems in contrast to the 3D HFM environment. The presence of the tight junction protein ZO-1 along the boundaries of the cells (Fig. 3d) further endorsed the epithelial character of a homogenous and polarized cell monolayer on HFM. In addition to monolayer polarization, the expression of OAT1, BCRP and MRP4 in ciPTEC was compared between 2D and 3D cultures. Interestingly, significantly increased expression levels of OAT1 were observed (Fig. 3e) as compared to flat monolayers and a trend towards an increase in BCRP and MRP4 (Fig. 3f,g) was shown, respectively. These data assume that a 3D environment induces membrane transporter expression, which might be the result of an improved epithelial character in 3D.

### Bioengineered renal tubules show organic anion transport activity

**Figure 4:**
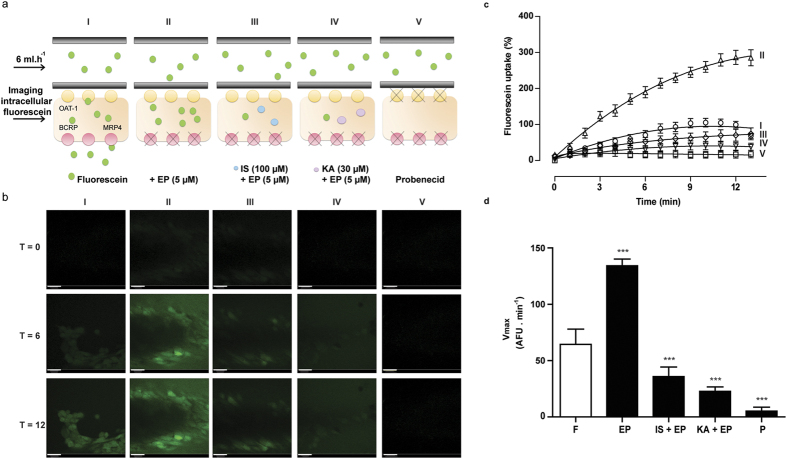
OAT1-mediated fluorescein uptake in bioengineered renal tubules. Fluorescein was used as a model substrate in an advanced fiber perfusion system. (**a**) Schematic presentation of the experimental set-up: I. Fluorescein perfusion, II. Fluorescein perfusion in the presence of efflux pump inhibitors (EP; (5 μM)), III and IV. Fluorescein perfusion in the presence of efflux pump inhibitors and indoxyl sulfate (III; 100 μM) or kynurenic acid (IV; 30 μM), V. Fluorescein perfusion in the presence of probenecid (P; 100 μM). (**b**) Representative real-time images of the five different conditions. Scale bare: 10 μm. (**c**) Semi-quantification of fluorescein uptake data in the absence or presence of the efflux pumps inhibitors solely, in combination with IS and KA, and in the presence of probenecid. From each single replicate 4 different regions in focus were analyzed. Data were fitted according to one-site total binding saturation curve using non-linear regression analysis. (**d**) Vmax values from all conditions were calculated from the corrected AFU data according to michaelis-menten kinetics using non-linear regression analysis. For statistical analysis, the Vmax values obtained from condition EP and P where compared to only fluorescein uptake (F). Conditions IS + EP and KA + EP were compared to EP. Data are shown as mean ± S.E.M. of three independent experiments performed in duplicate. ***p < 0.001, using one-way ANOVA followed by Dunnett’s multiple comparison test.

To study the activity of OAT1, BCRP and MRP4 in the bioengineered renal tubules, we used the substrate fluorescein and life confocal imaging (Fig. 4a,b). Perfusion of the tubes with fluorescein solely (I) resulted in an in intracellular fluorescent signal, which increased significantly in the presence of efflux pumps inhibitors (II). In the presence of indoxyl sulfate (III) and kynurenic acid (IV), the fluorescein uptake was inhibited resulting in a less intense intracellular fluorescent signal. In the final condition (V), fluorescein uptake was studied in the presence of probenecid, a blocker of OAT1, which resulted in a strongly diminished uptake. Semi-quantification of the normalized time-lapse experiment is presented in Fig. 4c. For statistical analysis, maximum uptake (V_max_) values were calculated from background corrected arbitrary fluorescence units (AFU) data (Fig. 4d). A significant increased fluorescein uptake was detected when studied in the presence of efflux pumps inhibitors (208 ± 10%; p < 0.001). Again, this was inhibited by indoxyl sulfate (45 ± 13%; p < 0.001) and kynurenic acid (83 ± 3%; p < 0.001). In the presence of probenecid, fluorescein uptake was clearly attenuated as well (96 ± 3%; p < 0.001). These findings are compatible with the formation of a functionally active bioengineered renal tubule.

### Bioengineered renal tubules facilitate uremic toxin secretion despite protein binding

Lastly, we investigated the capability of the bioengineered renal tubules to actively secrete protein bound uremic toxins. Active clearance of indoxyl sulfate and kynurenic acid in the absence of human serum albumin was studied by perfusing the tubules with 100 μM of the uremic toxin and measuring transport into the apical compartment. This revealed a clearance of 44 ± 6 μl.min^−1^.cm^−2^ and 72 ± 20 μl.min^−1^.cm^−2^ for indoxyl sulfate and kynurenic acid, respectively. This secretion was attenuated by probenecid (by 55 ± 6, p < 0.05 and 71 ± 7%, respectively) and by the efflux pumps inhibitors (68 ± 5, p < 0.001; 68 ± 6%, respectively), indicating active transepithelial transport of both uremic solutes across the epithelial cell monolayer. In a separate experiment we observed that clearance of the uremic toxins was fully restored after the probenecid treatment, when the bioengineered tubules were re-perfused with the toxins (data not shown). These findings confirmed that the cell monolayer is still viable after multiple treatments, including probenecid incubation, and that probenecid has an inhibitory effect on transport protein level solely. To further mimic the physiological situation, the ability of the bioengineered tubules to facilitate protein-bound toxin transport was shown. In this setup, we achieved a protein binding of indoxyl sulfate slightly lower than previously reported (73 ± 3%)^28^ in the presence of 1 mM HSA. Under the same conditions, kynurenic acid showed a protein binding of 63 ± 5%. Interestingly, the clearance of protein-bound indoxyl sulfate (74 ± 10 μl.min^−1^.cm^−2^, p < 0.01) and kynurenic acid (101 ± 23 μl.min^−1^.cm^−2^, ns) was enhanced when compared to the clearance of both toxins in the absence of albumin. Again, transport was attenuated by probenecid (99.4 ± 0.2, p < 0.001; 49 ± 15%, respectively) and by the efflux pumps inhibitors (98.9 ± 0.2, p < 0.001; 58 ± 14%, respectively). These findings suggest that protein binding positively affects the renal tubular clearance of the uremic toxins.

**Figure 5:**
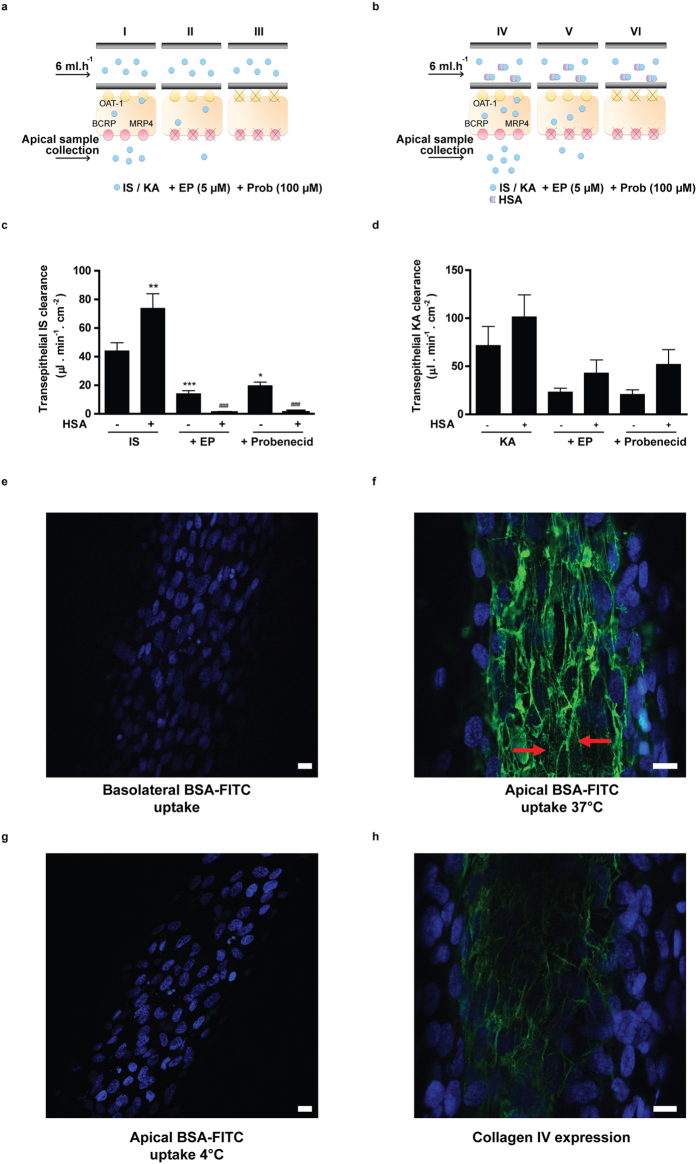
Transepithelial clearance of indoxyl sulfate and kynurenic acid in the presence of human serum albumin, and albumin-FITC handling in bioengineered renal tubules. (**a–d**) Transepithelial clearance of indoxyl sulfate and kynurenic acid. Schematic presentation of the experimental set up of IS and KA transepithelial transport in the absence (**a**) or presence (**b**) of HSA is reported. Quantification of IS (**c**) and KA (**d**) clearance in the absence (−) or presence (+) of HSA. The protein bound fraction of IS was 73 ± 5% and 63 ± 8% of KA was bound to albumin. Data are shown as mean ± S.E.M. of three independent experiments performed in duplicate. *p < 0.05, **p < 0.01 compared to IS in the absence of HSA, ^###^p < 0.001 compared to IS in the presence of HSA. Statistical analysis was performed using one-way ANOVA followed by Tukey’s multiple comparison test. (**e–g**) Endocytosis-mediated albumin uptake in bioengineered renal tubules. Cellular BSA-FITC uptake (green) in renal tubules (nuclei: blue) after 30 min exposure from the (**e**) basolateral compartment at 37 °C (**f** ) apical compartment at 37 °C and (**g**) apical compartment at 4 °C. Active uptake was detected solely when BSA-FITC uptake was performed at 37 °C, as indicated by the red arrows. (**h**) Collagen IV expression (green) in renal tubules showed a highly similar pattern as BSA-FITC uptake as shown in b. Scale bare: 10 μm.

To study the mechanism of protein bound uremic toxin handling further, we not only performed a basolateral perfusion with FITC-bovine serum albumin (BSA-FITC), but also studied the uptake of BSA-FITC from the apical site. Wilmer and Jansen *et al.* previously reported on the presence of a megalin-mediated albumin reabsorption mechanism in ciPTEC, located at the PTEC apical membrane^21^,^22^. Figure 5e–g shows BSA-FITC uptake in renal tubules after basolateral or apical exposures. Upon basolateral perfusion, intracellular BSA-FITC could not be detected (Fig. 5e). This observation is in agreement with the retention of albumin in the blood compartment under physiological conditions to prevent protein loss^29^. Figure 5f shows that active uptake of BSA-FITC was detectable when exposed to the apical compartment at 37 °C, thereby confirming that reabsorption is functional in renal tubules (red arrows). Importantly, this uptake was highly reduced when apical exposure took place at 4 °C (Fig. 5g), thereby further confirming the presence of an active endocytosis process in the bioengineered kidney tubules. Note that the BSA-FITC reabsorbed at 37 °C binds to collagens present in the tubules^27^. As described by Rueth *et al.*, albumin is known to bind hydrophobic compounds like collagen^30^. Concurrently, the pattern observed in ciPTEC upon BSA-FITC reabsorption indeed showed high similarities to the pattern observed when stained against collagen IV (Fig. 5h). As a consequence, the typical endocytosis BSA-FITC particles, which are usually detected in this reabsorption mechanism, are less clear in this setting. Nevertheless, these data show that albumin is actively reabsorbed by the apical membrane and not transported along with uremic toxins from the basal compartment across renal tubules.

## Discussion

The renal clearance of organic anions, including protein bound endogenous metabolites, highly depends on active secretion, which is confined to the tubular system. In ESRD, this function is severely compromised resulting in the accumulation of these metabolites in patients. Current dialysis therapies insufficiently remove the protein bound uremic toxins, which contributes to the high morbidity and mortality rates of the disease^31^, therefore, alternative treatment strategies are warranted. In this study, a bioengineered renal tubule was successfully developed and transepithelial transport of albumin-bound uremic toxins was demonstrated. Key in this process is the concerted action between (basolateral) uptake and (apical) efflux performed by transporters with designated membrane localization. In exchange for intracellular α-ketoglutarate, the OATs efficiently translocate organic anions from the blood compartment into the intracellular space against an electrochemical gradient. The efflux pumps belonging to the ABC family of transmembrane transporters then couple ATP hydrolysis to their urinary excretion in a unidirectional fashion. Using a unique, robust and complete human cell model, we first identified eight anionic uremic toxins for their interaction with OAT1- and OAT3-mediated transport in flat cell monolayers. Next, the essential role of the ABC transporters BCRP and MRP4 in cellular detoxification through efflux was demonstrated. Finally, a three-dimensional bioengineered tubule containing a polarized cell monolayer with a clear epithelial barrier function was developed. These human bioengineered tubules demonstrated active clearance of albumin-bound anionic uremic toxins as a next step in BAK engineering. Note that the formation of a cellular barrier is not unique for ciPTEC but also found for intestinal epithelial cells when cultured on HFM (data not shown).

The concept of BAK engineering was initiated in the late 80’s of last century by Aebischer *et al.* with non-human cell models (*i.e.* the canine derived MDCK or pig originating LLC-PK1 cells) cultured on HFM^32^. Follow-up studies by Humes and co-workers used cells of human origin, but with a major focus on the immunomodulatory effects for treatment of critically ill patients suffering from acute kidney injury^33^,^34^,^35^. Phase I and II clinical trials showed reduced cytokine levels and long-term survival improvement compared to the non-treated group. However, a follow-up clinical phase IIb trial failed due to difficulties in the manufacturing process of the BAK and the clinical study design^36^. One of the challenges to successfully develop a BAK is a suitable cell source to replace transport functions of the kidneys^34^,^37^. Primary renal epithelial cells show a high batch-to-batch variability in quality and function and dedifferentiation upon prolonged cultivation, and therefore hamper reproducibility. Moreover, to obtain a sufficient number of primary renal epithelial cells for a BAK approach is another hurdle to overcome^37^. Recent advances in the application of stem cells (i.e. of embryonic origin or induced pluripotent cells) into a PTEC-like phenotype is a promising alternative^38^,^39^,^40^, but this has not been characterized sufficiently nor their capability to maintain the OATs in culture has been proven. Human renal cells loose the expression of these essential transport proteins and (in part) their proximal tubular phenotype upon culturing^21^,^41^. This phenomenon has already been described in 1990^42^ and has, as of yet, not been solved, however, stable expression of OATs in renal cell lines is a prerequisite for renal functional replacement therapies as emphasized in a BAK. Here, we applied a recently optimized, robust and translational human renal cell model that endogenously expresses a panel of renal xenobiotic transporters successfully complemented with the OATs^21^,^22^,^23^. This appeared to be an asset, as we demonstrated for the first time active renal tubular secretion of the protein-bound uremic toxins indoxyl sulfate and kynurenic acid.

Using a conventional two-dimensional approach, the potency of eight uremic toxins to inhibit basolateral OAT1 and −3 mediated transport at clinically relevant concentrations was demonstrated^6^,^20^. The inhibitory potencies are in good agreement with previously published data obtained in animal models or in transfected cell systems expressing OAT1 or OAT3^18^,^20^. We selected two uremic toxins because of the highest inhibitory potency (kynurenic acid, this study) and widely described associations with uremic syndrome complications (indoxyl sulfate)^8^,^11^. Indoxyl sulfate has been considered to be a causative factor in CRF progression^43^, with most likely a dominant role of hOAT1 as its affinity for this transporter is at least 3-fold higher as compared to OAT3 (this study and^18^). Parent ciPTEC, lacking OAT1, were more resistant against indoxyl sulfate, but in ciPTEC-OAT1 a concentration-dependent reduction in cell viability was found. This effect was amplified when inhibitors of the efflux transporters, BCRP and MRP4, were applied, confirming the concerted action of uptake and efflux transporters in uremic toxin handling. Kynurenic acid showed a lower intrinsic toxicity as compared to indoxyl sulfate, despite its higher affinity for OAT1 (this study), BCRP and MRP4^14^. The molecular mechanisms through which both uremic toxins exert their cytotoxic potential are largely unrevealed.

The high affinity of kynurenic acid for OAT1, BCRP and MRP4 was demonstrated further by the higher bioengineered renal tubular clearance as compared to the elimination of indoxyl sulfate, either in the presence or absence of albumin. Moreover, it was shown that albumin itself is not taken up by ciPTEC from the basolateral site, but actively reabsorbed from the apical compartment. Interestingly, in the presence of albumin the transport of indoxyl sulfate and kynurenic acid was enhanced, which emphasizes the ability of ciPTEC to shift the protein-binding and allow for active secretion of the free fraction. These data support that albumin may stimulate transport of organic anions, as was suggested earlier in the 80’s of last century by Besseghir and Depner *et al.* They showed that albumin facilitated para-aminohippurate uptake in isolated rabbit proximal tubules and rat kidney slices^44^,^45^. Moreover, Pichette *et al.* demonstrated altered dynamics of furosemide in hypoalbuminaemic rabbits^46^,^47^. Furosemide is a known diuretic agent, and organic anion, which targets the Na^+^-K^+^-Cl^−^ cotransporter-2 (NKCC2) located downstream the proximal segment in the apical membrane of the thick ascending limb of Henle’s loop. Furosemide is highly protein bound (approx. 98%^46^,) and actively secreted via the proximal tubules into the apical compartment. In hypoalbuminaemic rabbits, higher furosemide doses were required to achieve a similar diuretic effect as in control rabbits^46^, supporting the important role of albumin in facilitating active transport of the organic anion. This may be the result of electronegative to -neutral transition of the compounds when bound to albumin and/or post-translational modifications of albumin when toxins are bound^48^,^49^. The tertiary structure of albumin may also stimulate the binding and transport of metabolites across the capillary wall into the interstitial compartments. As shown recently, the binding capacity of albumin was demonstrated to be diminished in CRF patients, most likely due to post-translational guanidylation of albumin sites^30^. The attenuated albumin binding capacity in CRF patients will probably contribute to less efficient transport of uremic toxins by PTEC, thus resulting in elevated plasma levels and their known consequences. Note that the double-coated HFM used in our bioengineered renal tubules allow albumin to reach the target transporters, emphasizing further the potential of the device in BAK applications. Future research should elucidate the impact of modified albumin as observed in uremic patients in the removal of uremic toxins using a BAK platform.

Altogether, a successful bioartifical renal tubule was established which presented a clear barrier function and facilitated transepithelial transport of protein-bound indoxyl sulfate and kynurenic acid. This provides an innovative basis for regenerative nephrology through advanced function replacement and paves the way to progress towards potential clinical applications focusing at: i) optimization and up-scaling of the system with the aim of maintaining the best possible cell function under sterile conditions for extended time periods; ii) *in vitro* validation of the bioreactor with respect to uremic toxin kinetics; iii) the safety aspects as ciPTEC are classified as GMO’s, for which thorough research in agreement with European guidelines for advanced therapy medicinal products (European Medicines Agency) is needed to enable the applications of GMO in medicinal products^50^; iv) preclinical validation of the model, and v) prediction of uremic toxin clearance by the bioengineered system in clinical settings, for which research should be directed at building a physiologically-based computational model. This allows predicting the capacity needed for treatment of uremia and defining the most suitable application strategies in function of solute kinetics. Hence, such model would not only apply to uremia treatment, but can be used for mimicking 3D scenarios of kidney disease modeling as well as for drug- toxicity and - efficacy testing.

## Methods

### Chemicals and cell culture materials

Chemicals were purchased from Sigma-Aldrich (Zwijndrecht, The Netherlands) unless stated otherwise. The uremic toxins *p*-cresylsulfate and *p*-cresylglucuronide were synthesized by the Institute for Molecules and Materials, Radboud University, Nijmegen, The Netherlands. MicroPES type TF10 hollow fiber capillary membranes (wall thickness 100 μm, inner diameter 300 μm, max pore size 0.5 μm) were obtained from Membrana GmbH (Wuppertal, Germany). Cell culture plates were purchased from Greiner Bio-One (Monroe, NC).

### Cell culture of ciPTEC-OAT1 and -OAT3

The transduction of OAT1 and OAT3 in ciPTEC^22^ was performed as previously described by Nieskens *et al.*^23^ and cells were cultured in supplemented PTEC culture media as described by Jansen *et al.*^21^. Fibers were coated and seeded as described by Jansen *et al.*^27^.

### Fluorescein inhibition assay

The potency of a panel of eight anionic uremic toxins to inhibit OAT1- and OAT3-mediated fluorescein uptake was investigated in flat monolayers using an inhibition assay as previously described by Nieskens *et al.*^23^. Blank fluorescence data were subtracted and relative data compared to control were plotted.

### Uptake of indoxyl sulfate and kynurenic acid by ciPTEC-OAT1

In short, active OAT1-mediated uptake of indoxyl sulfate and kynurenic acid was investigated using two concentrations of toxins (3 and 30 μM) in the presence or absence of probenecid (100 μM), kynurenic acid (100 μM) or indoxyl sulfate (100 μM). Intracellular toxin concentrations were analyzed using a LC-MS/MS system (Thermo scientific, Breda, The Netherlands) following the method described by Mutsaers *et al.*^14^. Data processing was performed using the Thermo Xcaliber software (Thermo scientific, version 2.1) and absolute data were plotted.

### Cell viability assay

An MTT (3-(4,5-dimethylthiazol-2-yl)-2,5-diphenyltetrazolium bromide) cell proliferation assay was performed as previously described by Nieskens *et al.*^23^. Background values were subtracted and normalized data were plotted.

### Monolayer polarization and transepithelial barrier function

To investigate the barrier function of matured ciPTEC-OAT1 cultured on HFM, fibers were perfused with FITC-inulin (0.1 mg/ml in Krebs-Henseleit buffer supplemented with 10 mM Hepes (KHH, pH 7.4)) and diffusion was measured in real-time. From each single replicate 4 different regions in focus were analyzed. Semi-quantification of real-time data was performed using Image J software (version 1.40 g) and normalized data were plotted. To determine the polarization of the ciPTEC-OAT1 monolayer on HFM, the expression of tight junction protein zonula occludens-1 (ZO-1) was investigated according to the protocol as previously described by Jansen *et al.*^27^.

### Detection of OAT1, BCRP and MRP4 mRNA expression

The mRNA expression of OAT1, BCRP and MRP4 was examined in ciPTEC-OAT1 when cultured in flat monolayers and as bioartificial renal tubules as previously described by Jansen *et al.*^21^. Gene expression levels were normalized to expression levels of the reference gene GADPH and were expressed as fold increase compared to matured cells in well plates.

### Fluorescein assay

To determine the OAT1 as well as BCRP and MPR4 transport activity in matured ciPTEC-OAT1 cultured on HFM, the fibers were connected to a similar perfusion set-up as was used for the barrier function assay. To measure active fluorescein uptake in real-time, fibers were perfused using 1 μM fluorescein in KHH in the presence or absence of specific drug transporter inhibitors or uremic toxins. Background corrected data were normalized against fluorescein uptake in the absence of inhibitors and were fitted according to one-site total binding saturation curve using non-linear regression analysis. In addition, V_max_ values (i.e. the maximum initial rate of a reaction) were calculated from the corrected AFU data according to Michaelis-Menten kinetics using non-linear regression analysis.

### Transepithelial transport of indoxyl sulfate and kynurenic acid

Transepithelial transport of indoxyl sulfate and kynurenic acid through the HFM with matured ciPTEC was studied using a similar perfusion set-up as was used for the barrier function assay. First, fibers were pre-incubated using efflux pump EP inhibitors (5 μM) or probenecid (100 μM) for 15 min. Next, the fibers were perfused using 100 μM IS or 30 μM KA in the presence or absence of inhibitors for 10 min and samples from the apical compartment were collected (100 μl). To determine the ability of ciPTEC to initiate a shift from the toxin protein-bound fraction to a free fraction for transport, similar conditions were investigated in the presence of human serum albumin ((HSA), 1mM). The binding efficiency of IS and KA to HSA was determined an ultrafiltration technique with a 30 kDA cut-off filter (Merck Millipore, Amsterdam, the Netherlands). The mixture was centrifuged for 15 min at 6,500 × rcf at rT and the ultrafiltrate containing the free toxin fraction was collected. The percentage albumin-bound (C_bound_) of indoxyl sulfate and kynurenic acid was calculated according to equation 1:


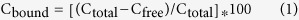


where C_total_ is the total concentration of toxin solution and C_free_ the free toxin concentration present in ultrafiltrate, both in μmol/l. All samples collected from the binding analysis and the apical compartment after the transport experiment were analyzed using a LC-MS/MS system as described earlier.

*In vitro* clearance values of the transepithelial transport of both toxins were calculated according to equation 2:


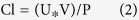


where U is the apical concentration in μmol/ml, V the volume in the apical compartment in ml and P the basolateral concentration in μmol/ml.

Next, the *in vitro* clearance was calculated according to equation 3:


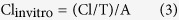


where Cl is the calculated clearance, T is the time in min and A the surface of the fiber in cm^2^.

All toxin samples were analyzed using a LC-MS/MS system as described earlier in this section.

### Endocytosis-mediated albumin uptake in bioengineered renal tubules

Matured renal tubules were assembled in our custom-made flow system and perfused with BSA-FITC (25 μg/ml) for 30 min at 37 °C. In the next condition, fibers were perfused with KHH buffer and BSA-FITC (25 μg/ml) was added apically and exposed for 30 min at 37 °C. As a control, the apical exposure was also performed at 4 °C in order to inhibit endocytosis. After the uptake was arrested, tubules were fixed using 2% (w/v) PFA for 5 min at room temperature. Finally, tubules were mounted using Prolong Gold Antifade Reagent with DAPI (Cell Signaling Technology, Leiden, The Netherlands) and BSA-FITC localization in the cells was examined using the Leica SPE-II – DM14000 (Leica Microsystems, Rijswijk, The Netherlands) and images were captured using the Leica Microsystems LAS-AF software version 1.00.71.

### Data analysis

All data are expressed as mean ± S.E.M of multiple replicates. Inhibition data were fitted according to one-site total binding saturation curve using non-linear regression analysis and Vmax values were calculated according to Michaelis-Menten kinetics using non-linear regression analysis. Statistical analysis was performed using one-way ANOVA analysis followed by Dunnett’s or Tukey’s multiple comparison test, or, when appropriate, an unpaired *t* test with GraphPad Prism version 5.02 (La Jolla, CA). A p-value of <0.05 was considered significant.

## Additional Information

**How to cite this article**: Jansen, J. *et al.* Bioengineered kidney tubules efficiently excrete uremic toxins. *Sci. Rep.* **6**, 26715; doi: 10.1038/srep26715 (2016).

## Supplementary Material

Supplementary Information

## Figures and Tables

**Table 1:** Uremic toxins inhibit OAT1- and OAT3-mediated fluorescein uptake.

Uremic toxin	C_m_ (μM) in ESRD patients^1^	ciPTEC-OAT1 (*IC*_50_ - μM)	R square	ciPTEC-OAT3 (*IC*_50_ - μM)	R square
Indoxyl sulfate	110	25 ± 4	0.92	83 ± 41	0.42
l-Kynurenine	6	65 ± 8	0.92	219 ± 66	0.54
Kynurenic acid	1	6 ± 1	0.95	6 ± 1	0.83
Indole-3-acetic acid	4	19 ± 2	0.93	148 ± 60	0.49
Hippuric acid	398	5 ± 1	0.95	22 ± 9	0.78
Indoxyl-β-glucuronide	9	492 ± 68	0.67	527 ± 218	0.55
*p*-Cresylglucuronide	44	2650 ± 922	0.28	588 ± 81	0.36
*p*-Cresylsulfate	675	79 ± 14	0.84	112 ± 19	0.80

The eight tested anionic uremic toxins inhibit OAT1- and OAT3-mediated fluorescein uptake in a concentration-dependent manner. Mean plasma levels of uremic toxins in ESRD patients were extracted from Duranton *et al.*^6^. R square: goodness of fit values extracted from non-linear regression analysis.
