# Supplementary material for: Bioengineered kidney tubules efficiently excrete uremic toxins
Source: Sci Rep. 2016 May 31;6:26715. doi: 10.1038/srep26715 (PMC4886219; doi:10.1038/srep26715)
Supplement: Supplementary Information [file srep26715-s1.pdf]

Bioengineered kidney tubules efficiently excrete uremic toxins

Supplementary material

Jansen J[1,2,3,4*], Fedecostante M[1,2,3,4*], Wilmer MJ[1], Peters JG[1], Kreuser UM[1], Bilos A[1], Mensink RA[5], Boltje TJ[5], Stamatialis D[6], Wetzels JF[7], van den Heuvel LP[3,8], Hoenderop JG[2], Masereeuw R[4†]

Department of [1]Pharmacology and Toxicology and [2]Physiology, Radboud university medical center, Radboud Institute for Molecular Life Sciences, Nijmegen, The Netherlands.

Department of [3]Pediatrics and [7]Nephrology, Radboud university medical center, Nijmegen, The Netherlands.

Division of [4]Pharmacology, Utrecht Institute for Pharmaceutical Sciences, Utrecht, The Netherlands..

Cluster for [5]Molecular Chemistry, Institute for Molecules and Materials, Radboud University, Nijmegen, The Netherlands.

Department of [6]Biomaterials Science and Technology, MIRA Institute for Biomedical Technology and Technical Medicine, University of Twente, The Netherlands.

Department of [8]Pediatric Nephrology & Growth and Regeneration, Catholic University Leuven, Leuven, Belgium.

[†] Corresponding author:

Rosalinde Masereeuw, Ph.D.

Utrecht University

Div. Pharmacology

Department of Pharmaceutical Sciences

Universiteitsweg 99

3584 CG Utrecht

The Netherlands

r.masereeuw@uu.nl

Phone: +31-30-253-3529

Fax: +31-30-253-7900

*both authors contributed equally.

Methods

Chemicals and cell culture materials

The uremic toxin p-cresylsulfate (pCS) was synthesized *via* a modified literature procedure [1]. NaOH was used instead of KOH to afford pCS as its sodium salt. ^{1}H-NMR (500 MHz, D_2O) δ 7.10 (d, *J* = 8.0 Hz, 1H), 6.78 (d, *J* = 8.0 Hz, 1H), 2.22 (s, 1H). ^{13}C-NMR (126 MHz, D_2O) δ 148.85, 136.49, 130.17, 121.33, 19.95. *p*-Cresylglucuronide (pCG) was synthesized according to a procedure by Desai and Blackwell *et al.* using a modified workup [2]. The crude product was purified by silica flash column chromatography (gradient: 0-15% H_2O/MeCN), after which the appropriate fractions were combined and concentrated *in vacuo*. The purified product was subsequently freeze dried to afford pCG as a white solid (60.5%). ^{1}H- and ^{13}C-NMR spectra matched the reported data.

Cell culture of ciPTEC-OAT1 and -OAT3

In standard cell culture approaches, cells were seeded in well plates or Transwell inserts using a density of 63,000 cells/cm^2 or 110,000 cells/cm^2 for ciPTEC-OAT1, respectively, and 82,000 cells/cm^2 for ciPTEC-OAT3 and were cultured for 24 h at 33°C, 5 % (v/v) CO_2, to proliferate and subsequently transferred to 37°C, 5 % (v/v) CO_2 for 7 days to mature.

Prior to ciPTEC-OAT1 seeding on the outside of HFM (1.0 x 10^6 cells/ml), fibers were biofunctionalized using a coating combination consisting out of 3,4-Dihydroxy-L-phenylalanine (L-DOPA; 2 mg/ml) and collagen IV (25 µg/ml), as previously described by Jansen *et al.* [27], and cells were seeded and cultured accordingly.

Fluorescein inhibition assay

Briefly, a concentration range (mM – nM) of indoxylsulfate, indole-3-acetic acid, indoxyl glucuronide, L-kynurenine, kynurenic acid, hippuric acid, p-cresylglucuronide and p-cresylsulfate were exposed to ciPTEC-OAT1 and -OAT3 cells in the presence of a known OAT substrate, fluorescein (1 µM), for 10 min at 37°C, 5 % (v/v) CO_2. After uptake arrest, intracellular fluorescence was detected at excitation wavelength 485 nm and emission wavelength 535 nm, using a Victor™ X3 multilabel platereader (Perkin-Elmer, Groningen, The Netherlands).

Uptake of indoxyl sulfate and kynurenic acid by ciPTEC-OAT1

Active OAT1-mediated uptake of indoxyl sulfate and kynurenic acid was investigated using two concentrations of toxins. Prior to the uptake, matured cells cultured in 48-well plates were pre-incubated using krebs-henseleit buffer supplemented with 10 mM Hepes (KHH buffer, pH 7.4) in the presence or absence of probenecid (100 µM), kynurenic acid (100 µM) or indoxyl sulfate (100 µM) for 30 min at 37°C, 5 % (v/v) CO_2. Next, the cells were exposed to either indoxyl sulfate (3 and 30 µM) or kynurenic acid (3 and 30 µM) for 10 min at 37°C, 5 % (v/v) CO_2 and the uptake was arrested using ice-cold KHH buffer. Finally, cells were lysed using perchloric acid (3.3 %

(v/v)) for 1h at room temperature (rT) and were ready for analysis.

Cell viability assay

Matured ciPTEC-OAT1 were exposed to a concentration range (mM - µM) of indoxyl sulfate or kynurenic acid in serum-free culture medium in the presence or absence of the BCRP and MRP4 efflux pump inhibitors, KO143 (10 µM) and MK571 (5 µM), for 24h at 37°C, 5 % (v/v) CO_2. After 4 hours of incubation, the intracellular accumulated precipitate was detected by measuring samples at a wavelength of 570 nm from which the background was subtracted, using a Benchmark Plus plate reader (Bio-rad Laboratories, Veenendaal, The Netherlands).

Monolayer polarization and transepithelial barrier function

To investigate the barrier function of matured ciPTEC-OAT1 cultured on HFM, the fibers were mounted on a separated inlet and outlet glass cannula (DMT Trading, Aarhus, Denmark) stabilized by a frame glued to a petri-dish, forming a separated basolateral (inner HFM) and apical compartment (outer HFM), containing KHH buffer (pH 7.4). A syringe pump (Terumo STC-521, Terumo Europe N.V., Leuven, Belgium) was connected to the inlet cannula by tubing, whereas the outlet cannula was connected by tubing to a depot to collect the perfusate. The chamber was installed on the Zeiss LSM510 META microscope (Zeiss, Oberkochen, Germany). Double coated HFM in the presence or absence of matured ciPTEC were perfused with FITC-inulin (0.1 mg/ml in KHH buffer) and diffusion was measured real-time for 13 min at 37°C, 5 % (v/v) CO_2.

Fixed and permeabilized cell monolayers were blocked and subsequently incubated against ZO-1(1:50 dilution in block solution; Invitrogen, Carlsbad, CA) for 1h at room temperature (rT). Next, cells were incubated with goat-anti-rabbit-Alexa488 conjugate (1:200, Abcam, Cambridge, UK) and finally nuclei were stained using DAPI (300 nM, Life Technologies) for 5 min at rT. Images were captured using the Olympus FV1000 Confocal Laser Scanning Microscope (Olympus, Tokyo, Japan) and the Olympus software FV10-ASW version 1.7.

Detection of OAT1, BCRP and MRP4 mRNA expression

To extract RNA from matured cells cultured on fibers, the RNAqueous® Micro Kit (Ambion, Carlsbad, CA) was used. RNA extraction from cells cultured in well plates was performed using the RNeasy® Mini Kit (Qiagen, Venlo, Netherlands), both kits were used according to the manufacturer's protocol. Subsequently, cDNA was synthesized using the M-MLV reverse transcriptase kit (Invitrogen, Carlsbad, CA). The mRNA expression levels of ciPTEC transporter genes were detected using gene specific primer-probe sets (Table S1) and TaqMan Universal PCR Master Mix (Applied Biosystems, Branchburg, NJ). The quantitative PCR reactions were performed using the CFX96 Real Time PCR system (Bio-Rad Laboratories, Veenendaal, Netherlands) and data were analyzed using the CFX Manager software (Bio-Rad Laboratories, Veenendal, Netherlands).

Fluorescein assay

To measure active fluorescein uptake, the fibers were perfused using 1 µM fluorescein in KHH in the presence or absence of specific drug transporter inhibitors (OAT1 inhibitor probenecid (100 µM), and efflux pump (EP) inhibitors KO143 (5 µM, to block BCRP), PSC833 (5 µM, to block any minor P-gp contribution) (Tocris Bioscience, Minneapolis, MN) and MK571 (5 µM, to block MRP4)) for 13 min at 37°C, 5 % (v/v) CO_2. Prior to fluorescein perfusion, fibers were pre-incubated using similar concentrations of inhibitors in KHH for 5 min at 37°C, 5 % (v/v) CO_2. To investigate the interaction of fluorescein (1 µM) and the uremic toxins indoxyl sulfate and kynurenic acid in ciPTEC cultured on HFM, fibers were exposed to indoxyl sulfate (100 µM) or kynurenic acid (30 µM) in combination with EP inhibitors for 13 min at 37°C, 5 % (v/v) CO_2. The fluorescein uptake was examined in real-time and imaging was performed using the Zeiss LSM510 META microscope (Zeiss, Oberkochen, Germany). Semi-quantification of real-time data was performed using Image J software (version 1.40g). From each single replicate 4 different cellular regions in focus were analyzed.

Methods to figure S1

Transepithelial barrier function of ciPTEC-OAT1 monolayers in 2D

To investigate the barrier function of matured ciPTEC-OAT1 cultured on 2D Transwell inserts, both compartments were washed once using KHH prior to FITC-inulin (0.1 mg/ml in KHH buffer) exposure basolaterally for 1h at 37°C, 5% (v/v) CO_2. Fluorescence was detected by measuring samples (75 µl) at excitation wavelength 485 nm and emission wavelength 535 nm, using a Victor™ X3 multilabel platereader. Blank data were subtracted and normalized data were plotted.

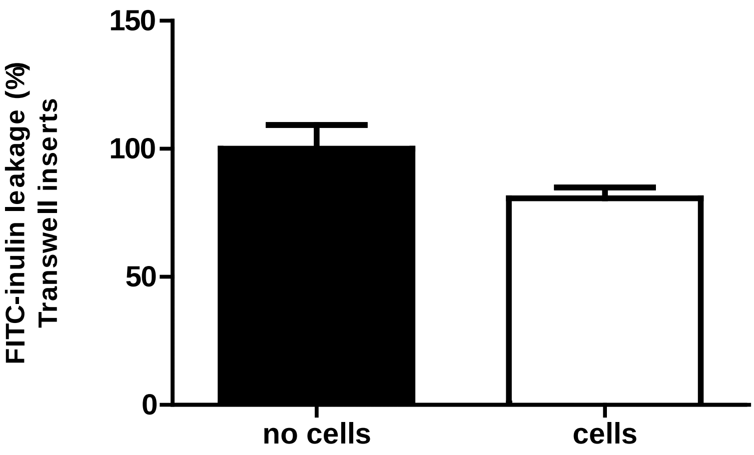

Figure S1. FITC-inulin diffusion in matured ciPTEC-OAT1 monolayers cultured in 2D systems. The FITC-inulin leakage was measured in matured ciPTEC-OAT1 seeded on coated Transwell® filter inserts. A limited transepithelial barrier function was observed in the presence of cells (white) compared to unseeded Transwell filters (black) after 13 min. Data are shown as mean ± S.E.M. of three independent experiments performed in triplicate.

Table S1: Taqman primer-probe sets.

Gene	Catalogue number
OAT1 (*SLC22A6*)	Hs00537914_m1
BCRP (*ABCG2*)	Hs00184979_m1
MRP4 (*ABCC4*)	Hs00195260_m1
GAPDH	Hs99999905_m1

References

1. Feigenbaum J, Neuberg CA. Simplified Method for the Preparation of Aromatic Sulfuric Acid Esters. *J Am Chem Soc.* **63,** 3529-3530 (1941).
2. Desai RN, Blackwell LF. TEMPO-mediated regiospecific oxidation of glucosides to glucuronides. *Synlett.* **13,** 1981-1984 (2003).
